# Identification of Macrophage Polarization-Related Genes as Biomarkers of Chronic Obstructive Pulmonary Disease Based on Bioinformatics Analyses

**DOI:** 10.1155/2021/9921012

**Published:** 2021-06-20

**Authors:** Yalin Zhao, Meihua Li, Yanxia Yang, Tao Wu, Qingyuan Huang, Qinghua Wu, Chaofeng Ren

**Affiliations:** Respiratory and Critical Care Medicine, Kunming First People's Hospital, Kunming, Yunnan Province, China

## Abstract

**Objectives:**

Chronic obstructive pulmonary disease (COPD) is characterized by lung inflammation and remodeling. Macrophage polarization is associated with inflammation and tissue remodeling, as well as immunity. Therefore, this study attempts to investigate the diagnostic value and regulatory mechanism of macrophage polarization-related genes for COPD by bioinformatics analysis and to provide a new theoretical basis for experimental research.

**Methods:**

The raw gene expression profile dataset (GSE124180) was collected from the Gene Expression Omnibus (GEO) database. Next, a weighted gene coexpression network analysis (WGCNA) was conducted to screen macrophage polarization-related genes. The differentially expressed genes (DEGs) between the COPD and normal samples were generated using DESeq2 v3.11 and overlapped with the macrophage polarization-related genes. Moreover, functional annotations of overlapped genes were conducted by Database for Annotation, Visualization and Integrated Discovery (DAVID) Bioinformatics Resource. The immune-related genes were selected, and their correlation with the differential immune cells was analyzed by Pearson. Finally, receiver operating characteristic (ROC) curves were used to verify the diagnostic value of genes.

**Results:**

A total of 4922 coexpressed genes related to macrophage polarization were overlapped with the 203 DEGs between the COPD and normal samples, obtaining 25 genes related to COPD and macrophage polarization. *GEM*, *S100B*, and *GZMA* of them participated in the immune response, which were considered the candidate biomarkers. *GEM* and *S100B* were significantly correlated with marker genes of B cells which had a significant difference between the COPD and normal samples. Moreover, *GEM* was highly associated with the genes in the PI3K/Akt/GSK3*β* signaling pathway, regulation of actin cytoskeleton, and calcium signaling pathway based on a Pearson correlation analysis of the candidate genes and the genes in the B cell receptor signaling pathway. PPI network analysis also indicated that *GEM* might participate in the regulation of the PI3K/Akt/GSK3*β* signaling pathway. The ROC curve showed that *GEM* possessed an excellent accuracy in distinguishing COPD from normal samples.

**Conclusions:**

The data provide a transcriptome-based evidence that *GEM* is related to COPD and macrophage polarization likely contributes to COPD diagnosis. At the same time, it is hoped that in-depth functional mining can provide new ideas for exploring the COPD pathogenesis.

## 1. Introduction

Chronic obstructive pulmonary disease (COPD), an inflammatory disease of the lung mainly caused by smoking tobacco cigarettes and environmental exposure from burning biomass fuels or air pollution [1, 2], has become a major health problem around the world [3]. It has been reported that COPD killed about 3 million people in 2016, and the mortality rates of COPD are still growing [4]. Worse, although COPD has long been considered treatable, the diagnosis and treatment of COPD in the clinical practice remain to be improved [5, 6]. Currently, the diagnosis of COPD mainly depends on the use of spirometry by identifying pulmonary dysfunction [7], which is full of limitations, such as the detection of the early stage of COPD. Moreover, although some blood-related biomarkers were related to exacerbations, progression, or mortality of COPD, it was unknown whether they could be selected as the diagnostic biomarkers [8]. On the other hand, even if the traditional treatments including lung volume reduction surgery for COPD have made great strides, there were still some uncontrollable complications [9]. In addition, even if some pulmonary rehabilitation programs, such as exercise training, education, and behavior change followed by patient-tailored therapies have been regarded as promising measures for improving the COPD patients, a few patients still cannot gain the benefit from the exercise training [10]. Hence, it is very wishful for further parsing the molecular mechanism and recognizing the novel biomarkers of COPD.

With the development of bioinformatics, recent studies have revealed that COPD susceptibility is associated with the genes' expression [11–14]. For example, it has been suggested that the expression of B3GNT, LAF4, and ARHGEF10 can predict the frequent exacerbations of COPD [11]. Moreover, IL6 and SOCS3 also have been suggested to play a key role in COPD and can be used as the therapeutic targets of COPD [12]. Moreover, Zhang et al. also found that TLR2 and CD79A may be used as the potential biomarkers for the clinical severity of COPD and related to the inflammatory responses of COPD [13]. More importantly, it has been revealed that COPD can be subdivided into three molecular subtypes based on the gene expression profiles of 213 COPD-related genes [14]. Therefore, bioinformatics analysis of gene expression profiling may contribute to screening novel biomarkers of COPD resulting in improving the treatment of COPD.

Increasing evidences have proposed that macrophage which is an important effector cell for the innate immune response plays a crucial role in COPD [15–17]. A recent study has demonstrated that COPD patients exhibit more macrophages in the bronchial alveolar lavage fluid, along the airways and lung parenchyma compared to the normal samples [15, 16]. Besides, Traves et al. also found more blood monocyte-derived macrophages into the airspaces of COPD [17]. More importantly, emerging evidences showed that macrophage polarization may be associated with COPD [18–20]. For example, a previous study has suggested an increase in proinflammatory M1 macrophages in the small airways of COPD compared to controls, but a reciprocal decrease in M2 macrophages [18]. On the other hand, the results of Eapen et al. suggested that lncRNA MIR155HG modulated the progression of COPD by inducing the granulocyte-macrophage colony-stimulating factor-mediated M1/M2 macrophage polarization [19]. In addition, Shaykhiev et al. suggested that the reprogramming for alveolar macrophage polarization likely contributes to COPD pathogenesis [20]. Nevertheless, the research focusing on the molecular regulation mechanism of macrophage polarization in COPD is inadequate.

With the development and extensive applications of bioinformatics, the weighted gene coexpression network analysis (WGCNA) has become an important and effective method to screen hub genes for complex disease [21]. In recent years, WGCNA has been performed to identify genes which are related to clinical features in many diseases, such as stroke [22] and schizophrenia [23]. Moreover, all of these researches suggested that screening biomarkers for diagnosis and treatment of complex disease by WGCNA was effective.

In the present study, we firstly downloaded the raw gene expression profile dataset which included COPD samples and normal samples from the Gene Expression Omnibus (GEO) database (https://www.ncbi.nlm.nih.gov/geo/query/acc.cgi). Next, WGCNA was carried out to screen macrophage polarization-related genes. Finally, we identified hub genes as the novel diagnostic biomarkers for COPD and further analyzed their molecular mechanisms in COPD, which will contribute to the treatment of COPD.

## 2. Materials and Methods

### 2.1. Dataset Acquisition

The raw gene expression profile dataset (GSE124180, only peripheral blood samples) was collected from the GEO database [24]. The dataset based on GPL16791 platform included 6 COPD samples and 15 normal samples.

### 2.2. WGCNA

The weighted gene coexpression network analysis was performed using WGCNA R package (v1.69) in the COPD and normal samples [25]. A total of 35 macrophage polarization genes (Supplementary Table 1) from the MsigDB database (https://www.gsea-msigdb.org/gsea/msigdb) were considered different traits to investigate the coexpressed genes related to macrophage polarization genes. Soft thresholding was then applied by raising correlation values to a power of 14 to amplify disparity between strong and weak correlations. The soft thresholding power was chosen to achieve approximately scale-free network topology, as recommended for biological networks [26, 27]. The resulting signed adjacency matrix was used to calculate topological overlap matrix (TO), which was then hierarchically clustered with (1-TO) as a distance measure.

Genes were then assigned into coexpression modules by dynamic tree cutting algorithm requiring minimal module size of 100 genes [28]. The modules with highly correlated eigengenes (correlation above 0.6) were merged. Module eigengene (ME) is the first principal component of the gene expression values within a module and is used to summarize the module's expression. The Pearson correlation between each gene and ME was then calculated. This value is called module membership (MM) and represents how close a particular gene is to a module. Finally, each gene was assigned to a module for which it had the highest MM. The module with the highest absolute value of the correlation coefficient with the traits was chosen as the key module for subsequent analysis.

### 2.3. Differential Expression Analysis and the Candidate Gene Identification

The comparison of differential expressions between the COPD and normal samples was generated using DESeq2 v3.11 [29]. The *p* value < 0.05 and ∣log_2_FC | >0.8 were considered the cutoff value. The candidate genes were identified by overlapping the DEGs and the genes in the key module, getting 25 genes related to COPD and macrophage polarization, and their expressions were displayed by a heatmap in the COPD and normal samples.

### 2.4. The Functional Annotations and Correlation Analysis of the Candidate Genes

DAVID bioinformatics resource (v6.8), an online website (https://david.ncifcrf.gov/), was devoted to conduct the functional annotations of the 25 genes [30]. The immune-related genes (as the candidate genes) were selected from the 25 genes, and Pearson analysis was used to investigate the correlation of the candidate genes and the differential immune cells between the COPD and normal samples, as well as marker genes and pathways of differential immune cells.

### 2.5. Construction of PPI Network and LASSO Model

To further evaluate the functions of the candidate genes, the PPI network was constructed by IPA (v01-18-05) to explore the gene-related pathways. Besides, a LASSO model was established by the candidate genes using the glmnet R package (v4.0-2) [31]. The ROC curve was drawn by the pROC R package (v1.16.12) and used to analyze the ability of the model and gene to distinguish the COPD and normal samples [32]. The higher the area under the curve (AUC), the stronger the predictive capacity.

## 3. Results

### 3.1. Selection of Coexpressed Genes Related to Macrophage Polarization

**Figure 1:**
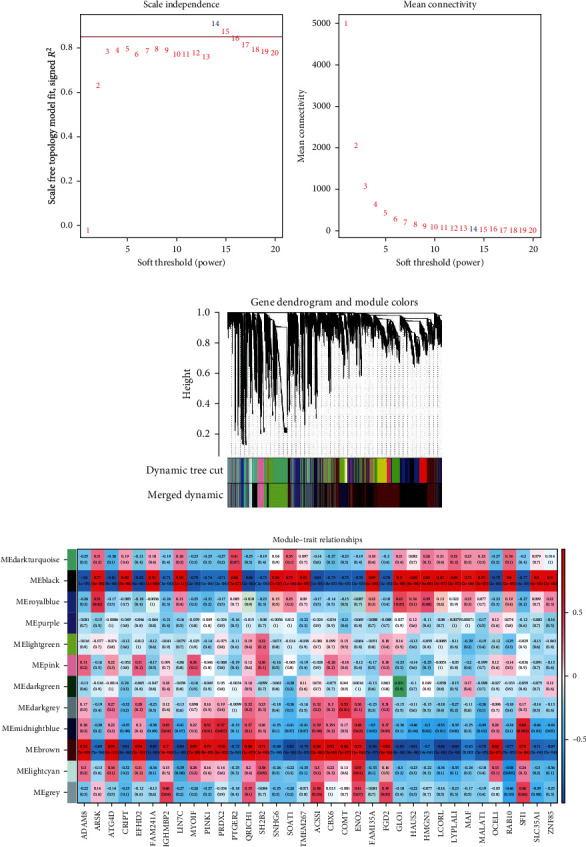
Construction weighted gene coexpression network and identification of modules related to the markers of macrophage polarization. (a) Determination the optimal soft threshold to conform to the scale-free distribution. (b) Dendrogram of genes clustered based on the highly correlated eigengenes (correlation above 0.6).

The coexpression network of the 21 samples was divided into 26 modules. We had chosen the soft threshold power 14 (marked with blue) to define the adjacency matrix based on the criterion of approximate scale-free topology (Figure 1(a)). The modules whose eigengenes were correlated above 0.6 would be merged (Figure 1(b)). Subsequently, the analysis of the relationship of the modules and the traits suggested that multiple modules (black, brown, and midnight blue) were closely related to one or more traits, especially the black module, which was the one with the highest correlation coefficient strongly associated with the macrophage polarization (Figure 1(c)). The black module contained 4922 eignegenes (Supplementary Table 2). Thus, the black module was selected as the coexpressed genes related to macrophage polarization.

### 3.2. Candidate Gene Identification

**Figure 2:**
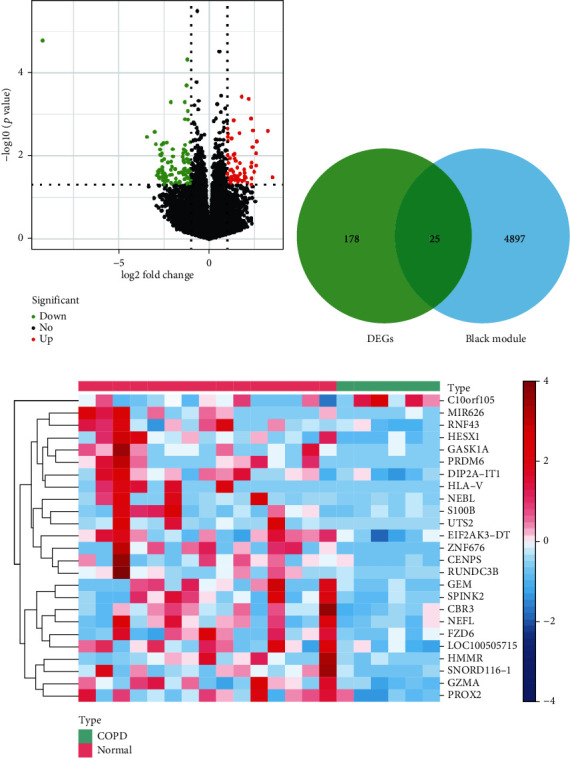
Identification of genes associated with COPD and macrophage polarization. (a) Volcano plot showed the DEGs between COPD samples and normal samples. (b) Overlapping genes between DEGs and macrophage polarization-related genes. (c) Heatmap exhibited the differential expression of the candidate genes between COPD samples and normal samples.

A total of 203 DEGs was generated between the COPD and normal samples and shown by a volcano plot (Figure 2(a)), including 82 upregulated genes (Supplementary Table 3) and 121 downregulated genes (Supplementary Table 4). The DEGs were overlapped with the 4922 eignegenes in the black module (Figure 2(b)), obtaining 25 genes as the candidate genes related to COPD and macrophage polarization. The candidate genes were listed in Supplementary Table 5, and their expression was shown by a heatmap which exhibited an obvious difference between the COPD and normal samples (Figure 2(c)). Almost all of the candidate genes in COPD samples had lower expressions compared to that in normal samples, except for *C10orf105* which was highly expressed in COPD samples, suggesting that it enabled these candidate genes related to macrophage polarization to commendably distinguish COPD from normal samples.

### 3.3. Functional Annotations of the Candidate Genes

To further explore the functions of the candidate genes, functional annotations were conducted by DAVID. Only 18 of the 25 candidate genes received corresponding functional annotations. As shown in Table 1, *GEM*, *S100B*, and *GZMA* participated in the immune response, which were considered the candidate genes for subsequent analyses. Macrophages are traditional innate immunocytes, as well as being involved into adaptive immune response. Macrophage polarization is a dynamic process, macrophages switch reversibly between M1 (proinflammatory) phenotype and M2 (anti-inflammatory) phenotype which is implicated in the immune response [33]. Therefore, we next would attempt to investigate the correlation between the candidate genes related to macrophage polarization and immunity.

It was worth noting that some genes were involved in the process of neuronal activity, such as *CBR3*, *FZD6*, *NEFL*, *PROX2*, *PRDM6*, *S100B*, and *GZMA*. *NEFL* was found to be involved in amyotrophic lateral sclerosis- (ALS-) related signaling pathways at the same time. Here, we speculated that when COPD occurred, these genes were regulated by macrophage polarization, which in turn affected motor neuron activity, resulting in motor neuron atrophy, and motor weakness and atrophy (like ALS). This may be the reason why patients with COPD often accompany unexplained skeletal muscle atrophy [34].

### 3.4. Relationship of the Candidate Genes with Immune Cells

**Figure 3:**
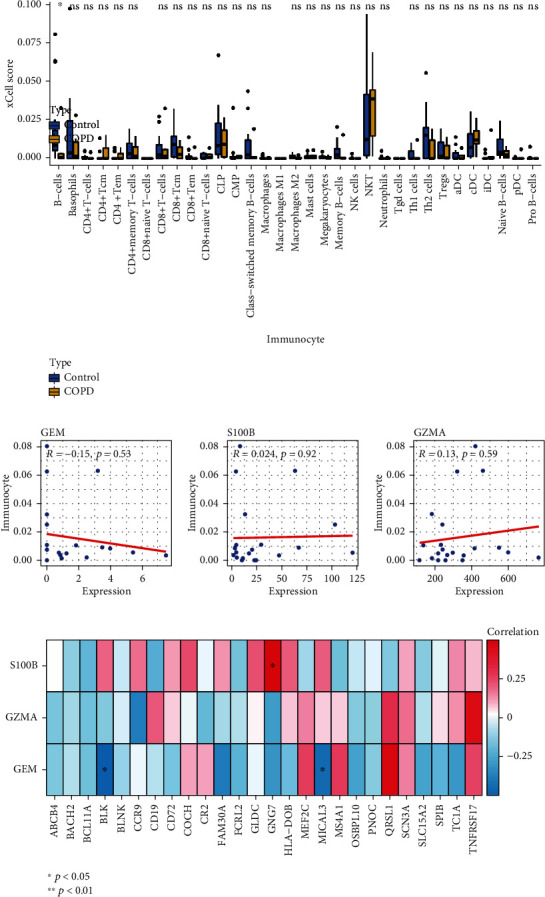
Correlation between candidate biomarkers and immune-infiltrated cells. (a) Box plot displayed the immune cell infiltration levels between COPD samples and normal samples. (b) Bubble plots showed the correlation between candidate biomarkers and immune-infiltrated cells. (c) Heatmap exhibited the correlation between candidate biomarkers and the marker genes of B cells.

To explore the correlation between the candidate genes and immunity, we analyzed the score of immune cells in the COPD and normal samples, showing that only the score of B cells had a significant difference between the COPD and normal samples. Compared with normal samples, score of the B cells was significantly reduced in the COPD samples (*p* value < 0.05, Figure 3(a)). But, *GEM*, *S100B* and *GZMA* were not significantly associated with the score of B cells (all *p* value > 0.05, Figure 3(b)), which might be caused by too small samples. To further study the correlation between the candidate genes and B cells, we downloaded the 26 marker genes from a literature with *Immunology Journal* [35]. Correlation of the candidate genes with the 26 marker genes were displayed by a heatmap (Figure 3(c)), suggesting that *GEM* was significantly negatively correlated with two marker molecules (*BLK* and *MICAL3*, all *p* values < 0.05), *S100B* was significantly positively correlated with one marker molecule (*GNG7*, *p* value < 0.05), while *GZMA* was not correlated with any marker molecules. Thus, it can be seen that *GEM* and *S100B* may participate in the progression of COPD via regulating activity of B cells.

### 3.5. Involvement of the Candidate Genes in the B Cell Receptor Signaling Pathway

**Figure 4:**
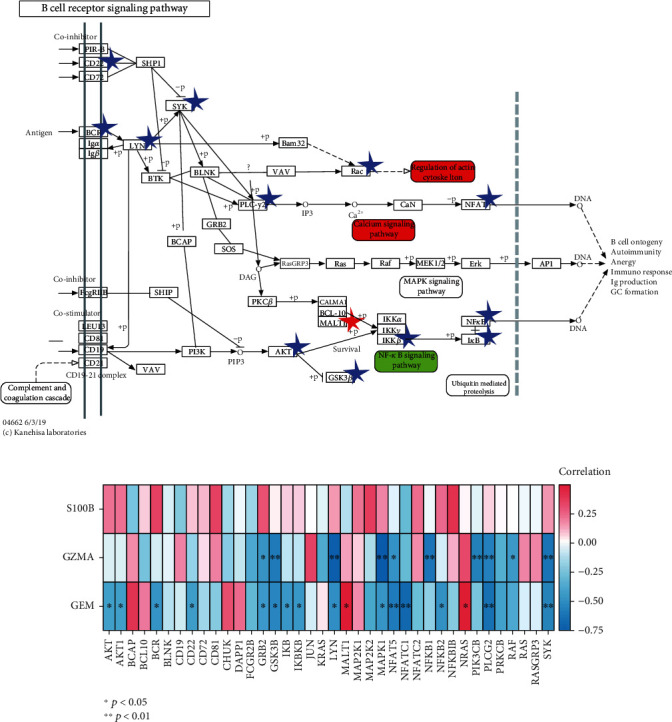
Association between candidate biomarkers and B cell receptor signaling pathway. (a) Regulation network of the B cell receptor signaling pathway. (b) Heatmap exhibited the correlation between candidate biomarkers and the genes in the B cell receptor signaling pathway.

The B cell receptor signaling pathway is of great importance for B cell survival and proliferation. The B cell receptor aberrantly expressed on B cells contributes to the multiple disease pathogenesis, and its signaling pathway is currently the target of several therapeutic strategies [36]. Therefore, we investigated the correlation of the candidate genes with the genes in the B cell receptor signaling pathway which were obtained from the Kyoto Encyclopedia of Genes and Genomes (KEGG) database (Figure 4(a)). The result indicated that *GEM* was highly associated with the genes in the PI3K/Akt/GSK3*β* signaling pathway (*AKT* and *GSK3B*, all *p* values < 0.05), regulation of the actin cytoskeleton (BCR, LYN, and RAC, all *p* values < 0.05), and the calcium signaling pathway (*SYK*, *PLCG2*, *NFAT5*, and *NFATC1*, all *p* values < 0.05) and might be partially related to the NF-*κ*B signaling pathway (only *MALT1*, *IKBKB, NFKB2*, and *IKB*, all *p* values < 0.05). Besides, no correlation between *GEM* and the MAPK signaling pathway was found (Figure 4(b)). Genes and pathways significantly related to *GEM* were marked in Figure 4(a). These results indicated that *GEM* might regulate B cell activity through the PI3K/Akt/GSK3*β* signaling pathway, regulation of actin cytoskeleton, and calcium signaling pathway, as well as the NF-*κ*B signaling pathway.

### 3.6. Construction of PPI Network

**Figure 5:**
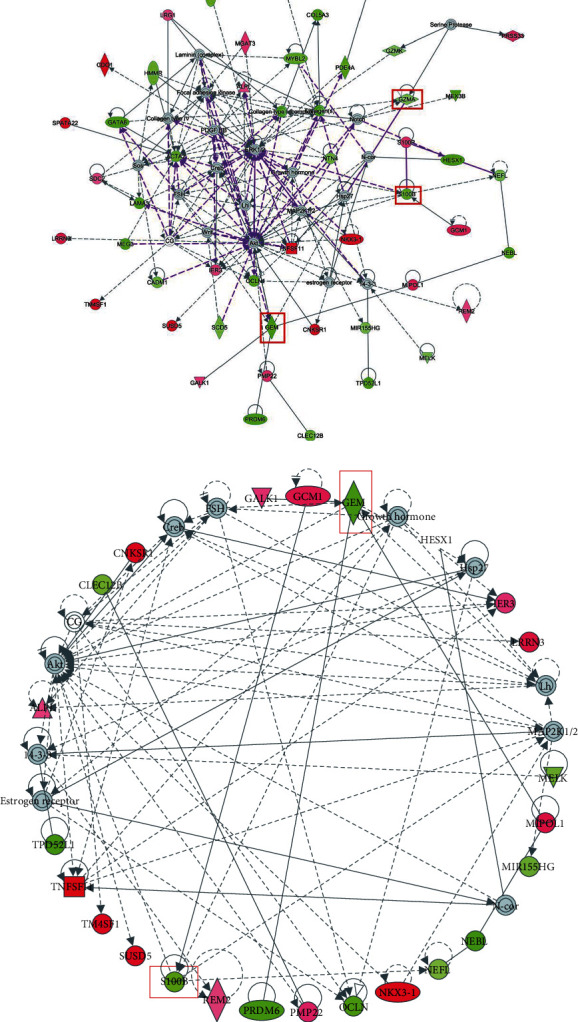
Construction of PPI network. (a) PPI network for candidate biomarkers and other DEGs. (b) PPI network for GEM and other DEGs.

To further verify *GEM*-regulated genes in pathways, all genes were used to construct a PPI network based on the screening of DEGs (Figure 5(a)), in which *GEM*, *S100B*, and *GZMA* were signed. In the network, red represents upregulated genes, and green represents downregulated genes. Gray indicates that the element is a predicted element. The solid line represents the proteins that have been experimentally confirmed to have physical contact with each other, and the dotted line represents that the interaction between the proteins is physically combined without being confirmed by the interaction experiment. The color of the line has no special meaning, and the arrow represents the object of action. Then, to show the interactions of *GEM* with other genes more clearly, we individually selected the PPI network of *GEM*. As shown in Figure 5(b), *GEM* was associated with Akt which was a gene that interacted with the most genes. This was consistent with our above results that *GEM* might participate in the regulation of the PI3K/Akt/GSK3*β* signaling pathway.

### 3.7. Diagnostic Value of *GEM*

**Figure 6:**
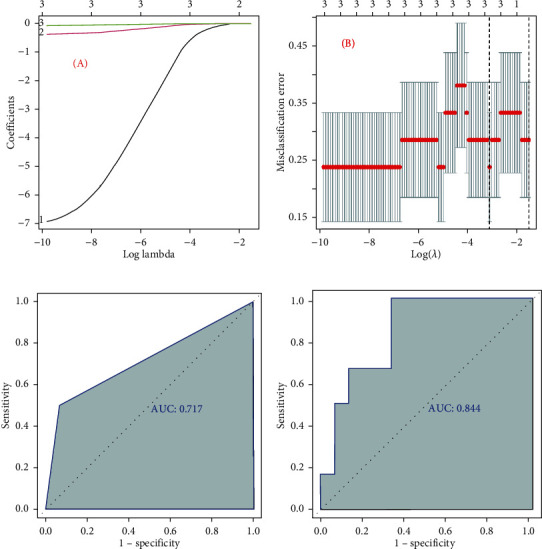
Investigation of the accuracy of the candidate biomarkers for distinguishing the COPD samples from normal samples. (a) Construction of the LASSO model based on 3 candidate biomarkers: (A) image showed the log (lambda) value of the 3 candidate biomarkers and (B) image showed the distribution of the log (lambda) value in the LASSO model. (c) ROC curve for the LASSO model. (d) ROC curve for the GEM.

To investigate the accuracy of the genes for distinguishing the COPD samples from normal samples, we firstly constructed a LASSO model using the three candidate genes (Figure 6(a)). The accuracy of the LASSO model was verified by the ROC curve, and the AUC was 0.717 (Figure 6(b)). Meanwhile, we plotted the other ROC curves with only *GEM*, and the AUC was 0.844 > 0.717 (Figure 6(c)). These results suggested that *GEM*, rather than the three candidate genes, possessed an excellent accuracy in distinguishing COPD from normal samples.

## 4. Discussion

COPD, characterized by persistent airflow obstruction, is an irreversible and preventable disease [37]. Although from 1990 to 2015, the death rate of COPD has gone down [38, 39], COPD will become the third leading cause of death worldwide in 2030 [40]. However, the diagnosis of COPD mainly relied on the spirometry values and clinical symptoms, which is full of continual underdiagnosis [41]. Hence, it is urgent to further study the molecular mechanism and screen novel and reliable biomarkers to improve the diagnosis of COPD.

Notably, there are some researches that focused on the GSE124180 dataset. For example, the authors uploaded the GSE124180 dataset mainly compared the correlation of gene expression across COPD-relevant tissues, including large-airway epithelium, alveolar macrophage, and peripheral blood, and explored the biological functions of overlapped genes among three tissues [42]. On the other hand, Baschal et al. compared the gene expressions across the lower airway, sinus, and middle ear tissues using the GSE124180 dataset and other data [43]. Different from these two researches, our study mainly focused on the role of macrophage polarization in COPD and identification of macrophage polarization-related genes as the biomarkers of COPD based on the peripheral blood of the GSE124180 dataset.

In our study, we identified 25 genes related to COPD and macrophage polarization, which were considered candidate genes for further analysis (Figure 2(b), Supplementary Table 5). Moreover, to further explore the functions of the 25 candidate genes, we performed the GO annotation and KEGG enrichment analyses. We found that for the biological process, these 25 candidate genes mainly involved in immune response, signal transduction, cell proliferation, negative regulation of skeletal muscle cell differentiation, and so on (Table 1). The analysis of KEGG pathway enrichment showed that these 25 genes mainly related to metabolic pathways, Wnt signaling pathway, Hippo signaling pathway, amyotrophic lateral sclerosis, ECM-receptor interaction, and so on (Table 1). Increasing evidence has revealed that obesity can affect the morbidity of COPD [44, 45]. Thus, we speculated that metabolic pathways may play an important role in COPD. In addition, it has been suggested that Wnt signaling is associated with the inflammatory response in lung alveolar epithelial cells [46]. Especially, in macrophages, Wnt5a can induce inflammatory response via FZD5 and Wnt3a can mediate anti-inflammatory effects by the Wnt/*β*-catenin signaling pathway [47]. Therefore, theses 25 genes might be associated with the COPD.

Clearly, the results of functional annotations for the 25 genes suggested that *GEM*, *S100B*, and *GZMA* participated in the immune response and were selected as the hub candidate genes (Table 1). *GEM*, a small GTP binding, is a member of RAS superfamily of monomeric G-proteins [48]. It has been suggested that *GEM* is involved in signal transduction [49]. Although it was not reported that *GEM* was related to COPD in recent studies, it has been suggested that *GEM* is related to the skeletal muscle for type 2 diabetes [49]. Thus, we speculated that GEM might be related to skeletal muscle dysfunction of COPD. *S100B*, a member of the *S100* protein family for Ca2^+^-binding proteins of the EF-hand type, is increasingly being studied in the central nervous system [50]. Moreover, a recent study has proved that *S100B* is associated with the reparative process in acute muscle injury and muscular dystrophy by regulating the M1/M2 macrophage levels [51]. In addition, it has been also found that *S100B* upregulated TNF-*α* and M1 markers in RAW264.7 macrophages [52]. *S100B* could participate in the FGFR1-mediated inflammatory response in osteoarthritis [53]. Besides, *S100B* was necessary for the progression of vascular immune responses in neonatally infected rat brains [54]. *GZMA*, a member of the serine protease family, may play a key role for the pathophysiology of different inflammatory disorders by acting as a proinflammatory mediator [55–57]. Garzón-Tituaña et al. also found that *GZMA* could serve as a proinflammatory mediator in macrophages by inducing the TLR4-dependent expression of IL-6 and TNF-*α* [58]. On the other hand, it has been reported that *GZMA* is involved in the COPD [59]. Notably, *S100B* and *GZMA* were associated with ALS-related signaling pathways. It has been demonstrated that muscle dysfunction is an important complication for COPD patients. Generally, COPD patients showed greater susceptibility muscle fatigue of the lower limb compared to healthy people [60–62]. Moreover, muscle mass loss or atrophy usually lead to the muscle dysfunction in COPD patients [34, 63]. Thus, *GEM*, *S100B*, and *GZMA* might be involved in the muscle dysfunction for COPD patients by ALS-related signaling pathways and might participate in the inflammatory response in COPD by regulating macrophage polarization, and these genes might act as the potential therapeutic targets for muscle atrophy of COPD patients.

Interestingly, *GEM* was significantly negatively correlated with *BLK* and *MICAL3*, which are the two marker molecules for B cells, and *S100B* was significantly positively correlated with the *GNG7* marker for B cells (Figure 3(c)). Moreover, we found that *GEM* was highly associated with the genes in the PI3K/Akt/GSK3*β* signaling pathway, regulation of actin cytoskeleton, and calcium signaling pathway and might be partially related to the NF-*κ*B signaling pathway (Figure 4(a)). Especially, *GEM* was related to the *Akt* in the PPI network (Figure 5(b)). These findings suggested that *GEM* and *S100B* might participate in the progression of COPD via regulating the activity of B cells and *GEM* might participate in the regulation of the PI3K/Akt/GSK3*β* signaling pathway. Apart from the macrophages, T lymphocytes also played an important role in COPD [64]. For example, Gosman et al. revealed that the number of B cells in bronchial biopsies of COPD was increased [65]. It has been revealed that glycyrrhizic acid could alleviate inflammatory lung disease, including chronic obstructive pulmonary disease by promoting the downstream PI3K/Akt/GSK3*β* signaling pathway [66]. Furthermore, compelling evidence has suggested that NF-*κ*B signaling plays a key role in the airway inflammation, including COPD [67]. Kim et al. also found that PI3K/Akt and NF-*κ*B signaling pathways were involved in the emphysematous change in COPD [68]. Taken together, these findings further suggested that *S100B* and *GEM* might serve as the therapeutic target and might become the significant targets for drug identification and drug designing of COPD.

Finally, we found *GEM* could more precisely distinguish COPD from normal samples than the LASSO model obtained by combined *GEM*, *S100B*, and *GZMA* (Figure 6(c)). Hence, GEM could act as an independent diagnostic biomarker for COPD.

## 5. Conclusions

In summary, our study for the first time analyzes the role of macrophage polarization-related genes in COPD by WGCNA. In the present study, we found that the *GEM*, *S100B*, and *GZMA* might be the novel therapeutic targets for COPD and *GEM* could act as an independent diagnostic biomarker for COPD. Our findings may contribute to further understanding the molecular mechanism and improving the clinical diagnosis and treatment for COPD. However, there are still many limitations in this study, for instance, sample limitation and experiment limitation. Therefore, further experimental studies are essential for further verifying the functions and mechanisms of *GEM*, *S100B*, and *GZMA* in COPD.

## Figures and Tables

**Table 1:** The results of GO functional annotation and KEGG pathways for the 25 candidate genes.

ID	Biological process	KEGG pathway
CBR3	GO:0042376 ~ phylloquinone catabolic process, GO:0050890 ~ cognition, GO:0055114 ~ oxidation-reduction process	hsa00590: arachidonic acid metabolism, hsa00980: metabolism of xenobiotics by cytochrome P450, hsa01100: metabolic pathways
FZD6	GO:0001736 ~ establishment of planar polarity, GO:0001843 ~ neural tube closure, GO:0001942 ~ hair follicle development, GO:0007186 ~ G-protein coupled receptor signaling pathway, GO:0007223 ~ Wnt signaling pathway, calcium modulating pathway, GO:0007275 ~ multicellular organism development, GO:0030168 ~ platelet activation, GO:0033278 ~ cell proliferation in midbrain, GO:0035567 ~ non-canonical Wnt signaling pathway, GO:0035880 ~ embryonic nail plate morphogenesis, GO:0042472 ~ inner ear morphogenesis, GO:0043433 ~ negative regulation of sequence-specific DNA binding transcription factor activity, GO:0060071 ~ Wnt signaling pathway, planar cell polarity pathway, GO:0090090 ~ negative regulation of canonical Wnt signaling pathway, GO:1904693 ~ midbrain morphogenesis	hsa04310: Wnt signaling pathway, hsa04390: Hippo signaling pathway, hsa04550: signaling pathways regulating pluripotency of stem cells, hsa04916: melanogenesis, hsa05166:HTLV-I infection, hsa05200: pathways in cancer, hsa05205: proteoglycans in cancer, hsa05217: basal cell carcinoma
NEFL	GO:0000165 ~ MAPK cascade, GO:0000226 ~ microtubule cytoskeleton organization, GO:0008089 ~ anterograde axonal transport, GO:0008090 ~ retrograde axonal transport, GO:0009636 ~ response to toxic substance, GO:0014012 ~ peripheral nervous system axon regeneration, GO:0019896 ~ axonal transport of mitochondrion, GO:0021510 ~ spinal cord development, GO:0021766 ~ hippocampus development, GO:0021987 ~ cerebral cortex development, GO:0031133 ~ regulation of axon diameter, GO:0033693 ~ neurofilament bundle assembly, GO:0040011 ~ locomotion, GO:0043434 ~ response to peptide hormone, GO:0043524 ~ negative regulation of neuron apoptotic process, GO:0043547 ~ positive regulation of GTPase activity, GO:0045105 ~ intermediate filament polymerization or depolymerization, GO:0045109 ~ intermediate filament organization, GO:0048812 ~ neuron projection morphogenesis, GO:0050772 ~ positive regulation of axonogenesis, GO:0050885 ~ neuromuscular process controlling balance, GO:0051258 ~ protein polymerization, GO:0051412 ~ response to corticosterone, GO:0061564 ~ axon development, GO:1903935 ~ response to sodium arsenite, GO:1903937 ~ response to acrylamide	hsa05014: amyotrophic lateral sclerosis (ALS),
ZNF676	GO:0006351 ~ transcription, DNA-templated, GO:0006355 ~ regulation of transcription, DNA-templated	
PROX2	GO:0000122 ~ negative regulation of transcription from RNA polymerase II promoter, GO:0006351 ~ transcription, DNA-templated, GO:0030182 ~ neuron differentiation, GO:0045944 ~ positive regulation of transcription from RNA polymerase II promoter, GO:0055007 ~ cardiac muscle cell differentiation	
HMMR	GO:0000086 ~ G2/M transition of mitotic cell cycle, GO:0030214 ~ hyaluronan catabolic process	hsa04512: ECM-receptor interaction
GEM	GO:0006955 ~ immune response, GO:0007067 ~ mitotic nuclear division, GO:0007165 ~ signal transduction, GO:0007166 ~ cell surface receptor signaling pathway, GO:0007264 ~ small GTPase mediated signal transduction, GO:0051276 ~ chromosome organization, GO:0051310 ~ metaphase plate congression	
HESX1	GO:0006351 ~ transcription, DNA-templated, GO:0007420 ~ brain development,GO:0030916 ~ otic vesicle formation, GO:0043584 ~ nose development, GO:0045892 ~ negative regulation of transcription, DNA-templated, GO:0048853 ~ forebrain morphogenesis,	hsa04550: signaling pathways regulating pluripotency of stem cells
PRDM6	GO:0006351 ~ transcription, DNA-templated, GO:0022008 ~ neurogenesis, GO:0034968 ~ histone lysine methylation, GO:0045892 ~ negative regulation of transcription, DNA-templated, GO:0051151 ~ negative regulation of smooth muscle cell differentiation	
*RUNDC3B*		
S100B	GO:0007409 ~ axonogenesis, GO:0007417 ~ central nervous system development, GO:0007611 ~ learning or memory, GO:0007613 ~ memory, GO:0008283 ~ cell proliferation, GO:0008284 ~ positive regulation of cell proliferation, GO:0008360 ~ regulation of cell shape, GO:0043065 ~ positive regulation of apoptotic process, GO:0043123 ~ positive regulation of I-kappaB kinase/NF-kappaB signaling, GO:0045087 ~ innate immune response, GO:0048168 ~ regulation of neuronal synaptic plasticity, GO:0048708 ~ astrocyte differentiation, GO:0051384 ~ response to glucocorticoid, GO:0051597 ~ response to methylmercury, GO:0060291 ~ long-term synaptic potentiation, GO:0071456 ~ cellular response to hypoxia, GO:2001015 ~ negative regulation of skeletal muscle cell differentiation	
CENPS	GO:0000712 ~ resolution of meiotic recombination intermediates, GO:0006281 ~ DNA repair, GO:0006312 ~ mitotic recombination, GO:0006974 ~ cellular response to DNA damage stimulus, GO:0007062 ~ sister chromatid cohesion, GO:0007067 ~ mitotic nuclear division, GO:0031297 ~ replication fork processing, GO:0031398 ~ positive regulation of protein ubiquitination, GO:0034080 ~ CENP-A containing nucleosome assembly, GO:0036297 ~ interstrand cross-link repair, GO:0051301 ~ cell division, GO:0051382 ~ kinetochore assembly	hsa03460: Fanconi anemia pathway
*C10orf105*		
GZMA	GO:0006915 ~ apoptotic process, GO:0006955 ~ immune response, GO:0019835 ~ cytolysis, GO:0032078 ~ negative regulation of endodeoxyribonuclease activity, GO:0043065 ~ positive regulation of apoptotic process, GO:0043392 ~ negative regulation of DNA binding, GO:0051354 ~ negative regulation of oxidoreductase activity, GO:0051603 ~ proteolysis involved in cellular protein catabolic process	hsa04080: neuroactive ligand-receptor interaction
NEBL	GO:0071691 ~ cardiac muscle thin filament assembly	
RNF43	GO:0016055 ~ Wnt signaling pathway, GO:0016567 ~ protein ubiquitination, GO:0030178 ~ negative regulation of Wnt signaling pathway, GO:0038018 ~ Wnt receptor catabolic process, GO:0042787 ~ protein ubiquitination involved in ubiquitin-dependent protein catabolic process, GO:0072089 ~ stem cell proliferation,	
SPINK2	GO:0002176 ~ male germ cell proliferation, GO:0007286 ~ spermatid development, GO:0009566 ~ fertilization, GO:0043065 ~ positive regulation of apoptotic process, GO:0060046 ~ regulation of acrosome reaction, GO:0072520 ~ seminiferous tubule development, GO:1900004 ~ negative regulation of serine-type endopeptidase activity	
UTS2	GO:0001666 ~ response to hypoxia, GO:0003105 ~ negative regulation of glomerular filtration, GO:0006936 ~ muscle contraction, GO:0007204 ~ positive regulation of cytosolic calcium ion concentration, GO:0007268 ~ chemical synaptic transmission, GO:0008217 ~ regulation of blood pressure, GO:0010459 ~ negative regulation of heart rate, GO:0010460 ~ positive regulation of heart rate, GO:0010763 ~ positive regulation of fibroblast migration, GO:0010841 ~ positive regulation of circadian sleep/wake cycle, wakefulness, GO:0032224 ~ positive regulation of synaptic transmission, cholinergic, GO:0032967 ~ positive regulation of collagen biosynthetic process, GO:0033574 ~ response to testosterone, GO:0035811 ~ negative regulation of urine volume, GO:0035814 ~ negative regulation of renal sodium excretion, GO:0042312 ~ regulation of vasodilation, GO:0042493 ~ response to drug, GO:0045597 ~ positive regulation of cell differentiation, GO:0045766 ~ positive regulation of angiogenesis,GO:0045776 ~ negative regulation of blood pressure, GO:0045777 ~ positive regulation of blood pressure, GO:0045909 ~ positive regulation of vasodilation, GO:0046005 ~ positive regulation of circadian sleep/wake cycle, REM sleep,GO:0046676 ~ negative regulation of insulin secretion,GO:0048146 ~ positive regulation of fibroblast proliferation,	

## Data Availability

The GSE124180 dataset analyzed in this study was available in the GEO database.

## Supplementary Materials

Supplementary Materials Supplementary Table 1: 35 macrophage polarization marker genes from the MsigDB database. Supplementary Table 2: 4922 genes in black module. Supplementary Table 3: 82 upregulated genes in COPD samples compared to normal samples. Supplementary Table 4: 121 downregulated genes in COPD samples compared to normal samples. Supplementary Table 5: overlapping genes between DEGs and macrophage polarization-related genes.
